# An Efficient Micropropagation Protocol for *Camellia chekiangoleosa* ‘Ganhongyou 1’ via Stem Segment Culture

**DOI:** 10.3390/plants15060871

**Published:** 2026-03-11

**Authors:** Anni Liu, Yixuan Peng, Xin Chen, Qiangqiang Cheng, Kang Zha, Qiang Wen

**Affiliations:** 1 College of Forestry, Jiangxi Agricultural University, Nanchang 330108, China; 13870877501@163.com (A.L.); chenxin20010705@163.com (X.C.); 2 Jiangxi Provincial Key Laboratory of Oil-Tea Camellia Resource Cultivation and Utilization, Jiangxi Academy of Forestry, Nanchang 330032, China; 18811716268@163.com (Y.P.); 1203ccw@163.com (Q.C.); 18715605056@163.com (K.Z.)

**Keywords:** *Camellia chekiangoleosa*, adventitious root primordia, plant growth regulators, ex vitro rooting, woody plant micropropagation

## Abstract

The provincial-level registered superior cultivar *Camellia chekiangoleosa* ‘Ganhongyou 1’ boasts superior economic traits coupled with significant ornamental value, driving demand for an efficient propagation system. Consequently, this study aimed to develop a rapid micropropagation protocol by investigating culture conditions using semi-woody nodal segments with axillary buds as explants on Hyponex basal medium supplemented with varying combinations of plant growth regulators. Contamination was effectively minimized to 18% by a combined approach of surface sterilization (75% ethanol, 0.1% HgCl_2_, and 20% NaClO) and incorporating 1 mL/L bactericide into the induction medium. For bud induction, the optimal medium was 2 g/L Hyponex supplemented with 1.0 mg/L 6-benzylaminopurine (6-BA) and 0.2 mg/L indole-3-acetic acid (IAA), achieving an 86.67% induction rate. The best proliferation was achieved on the medium containing 2 g/L Hyponex, 1.0 mg/L 6-BA, 0.15 mg/L 3-indolebutyric acid (IBA), and 0.5 mg/L gibberellic acid (GA_3_), yielding a proliferation coefficient of 6.53. A combined strategy, integrating in vitro pre-culture with ex vitro treatment, proved most effective for rooting and acclimatization: shoots were first pre-cultured for 20 days on 1/2 strength Hyponex medium supplemented with 0.5 mg/L 1-naphthaleneacetic acid (NAA) and 2.0 mg/L IBA, followed by ex vitro base treatment with 1.0 g/L ABT (a rooting powder complex) solution before transplantation into seedling bags. This approach resulted in an 88% survival rate. Furthermore, anatomical analysis revealed the origin of adventitious root primordia from phloem parenchyma cells, thereby confirming a phloem-rooting pattern for this species. In conclusion, this study establishes a practical and efficient micropropagation protocol for ‘Ganhongyou 1’, providing a reliable technical foundation for its commercial-scale seedling production.

## 1. Introduction

Oil-tea camellia, such as *Camellia oleifera* is one important woody oil crop, and its oil is rich in unsaturated fatty acids and various vitamins, which has been promoted by the Food and Agriculture Organization (FAO) as a healthy edible oil [1]. *C. chekiangoleosa* is one of the primary oil-tea camellia species in China. As an endemic species distributed in the mountainous regions of southern China, its seed oil surpasses the widely cultivated *C. oleifera* in several key aspects: extraction yield, content of unsaturated fatty acids (particularly oleic acid), and levels of natural antioxidants such as squalene and phytosterols [2,3,4]. Consequently, it possesses significant development potential for applications in the food, pharmaceutical, and cosmetic industries [5,6,7,8]. Despite these merits, conventional breeding of oil-tea camellia species, including *C. chekiangoleosa*, nonetheless faces significant constraints due to high heterozygosity and a long reproductive cycle, which lead to a shortage of superior germplasm resources [4,8]. Consequently, this bottleneck severely hampers varietal improvement and the sustainable development of the industry.

Plant tissue culture (PTC) serves as a pivotal biotechnology, playing an irreplaceable role in elucidating plant growth and development mechanisms, identifying gene functions, and creating transgenic plants [9,10,11,12]. It also provides a controlled experimental platform for germplasm conservation and efficient regeneration [13,14]. The successful establishment of this technical system highly depends on the appropriate selection of basal media and the precise regulation of plant growth regulators [14,15]. The type, concentration, and ratio of plant growth regulators directly determine the success or failure of key stages such as explant dedifferentiation, redifferentiation, and rooting [16]. The tissue culture of oil-tea camellia poses particular challenges. Its tissues are rich in polyphenols, making them prone to browning under in vitro conditions, and they exhibit high sensitivity to exogenous plant growth regulators, which therefore require careful screening and optimization [17]. Current studies mostly use buds or stem segments of oil-tea camellia as explants. These are cultured on basal media such as Murashige and Skoog medium (MS), woody plant medium (WPM), Hyponex, or 1/2 MS, supplemented with various combinations of cytokinins—for example, 6-benzylaminopurine (6-BA), benzyladenine (BA), zeatin (ZT), or thidiazuron (TDZ)—and auxins such as 1-naphthaleneacetic acid (NAA), indole-3-butyric acid (IBA), or indole-3-acetic acid (IAA) [18]. To improve culture outcomes, gibberellic acid (GA_3_) at concentrations ranging from 1.0 to 6.0 mg/L or casein hydrolysate at 500–600 mg/L is sometimes added as a supplement [19]. However, the rooting of shoots remains a critical bottleneck. Research indicates that IBA is widely used due to its high efficacy in inducing root primordia formation, and it can promote rooting either alone or in combination with NAA [20]. As conventional rooting media often yield limited results, a two-step method is frequently adopted: root primordia are first induced in a high-concentration auxin medium, followed by transfer to a hormone-free medium for further development [21], or alternatively, auxin soaking combined with ex vitro rooting is employed to improve efficiency [22]. Nonetheless, existing protocols still suffer from insufficient stability and reproducibility, and the responses to growth regulators vary significantly among different species and genotypes, which constrains the generalizability and broader application of the technique. Therefore, for *C. chekiangoleosa*, which has a relatively weak research foundation, systematically elucidating its response patterns to growth regulators and establishing an optimized, tailored system are of great significance for achieving efficient micropropagation and promoting its industrial application.

While micropropagation protocols have been developed for *C. oleifera*, no such system exists for *C. chekiangoleosa*. To address this gap, this study utilized nodal segments with buds of the superior cultivar ‘Ganhongyou 1’ to establish a complete micropropagation protocol. Key techniques including shoot induction, multiplication, and efficient rooting were systematically investigated. The aim was to develop a reliable in vitro propagation system, thereby providing a technical foundation for the large-scale production of high-quality planting material.

## 2. Results

### 2.1. Contamination Control with Different Disinfection Treatments

To establish an effective sterilization protocol, we evaluated seven different disinfection methods (i–vii). When explants were treated with 0.1% HgCl_2_ (method i) or 20% NaClO (method ii) for 7–10 min after 75% C_2_H_5_OH immersion, the contamination rate still exceeded 90% (Table 1). Although approximately 1% of the explants showed axillary bud swelling, their leaves failed to unfold; the remaining explants mostly remained unchanged or turned brown and died. When a pretreatment procedure was added (method iii)—repeatedly wiping stems with 75% ethanol and then rinsing under running water for 2–3 h—it not only lowered the contamination rate to 51% but also significantly increased the induction rate to 15.67%. Further reductions in contamination were achieved by additional measures: indoor pre-cultivation (method iv), an extra round of HgCl_2_ disinfection (method v), or an additional NaClO treatment (method vi). Among these, the supplementary NaClO treatment (method vii) was most effective, lowering the contamination rate to 24.67%. Building on method vii, a further and significant reduction in contamination—to 18.00%—was achieved by incorporating 1 mL/L bactericide into the induction medium. This combined approach was designated as method vii. Moreover, this combined approach not only achieved lower contamination but also markedly improved the bud induction rate and shoot vigor. The induced shoots exhibited robust growth, obvious elongation, long internodes, and numerous axillary buds—traits that are highly conducive to subsequent proliferation.

### 2.2. Axillary Bud Induction Using Different Media Formulations

To determine the optimal basal medium concentration, explants were cultured on Hyponex at varying levels (0.5, 1.0, 1.5, 2.0, 2.5, 4.0 g/L). At the lower end (0.5 g/L), nutrient insufficiency resulted in the lowest induction rate and poor bud development. Growth status improved with increasing concentration up to 2.0 g/L, which yielded the best outcomes. At the higher end (4.0 g/L), however, adverse effects including bud necrosis and leaf yellowing were observed (Table 2). Considering both the induction rate and growth performance, a Hyponex concentration of 2.0 g/L is determined to be optimal.

### 2.3. Axillary Bud Induction with Different PGR Combinations

**Figure 1:**
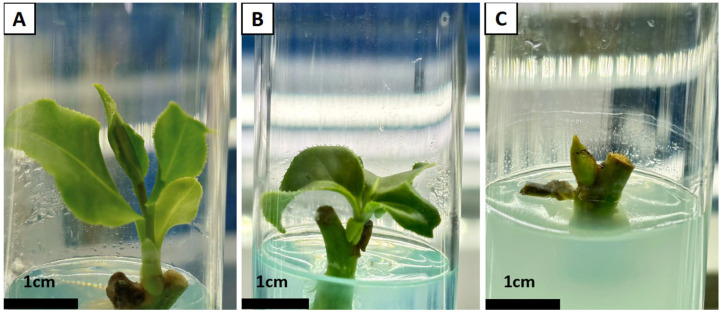
Growth induced by different hormones on bud segments. (**A**) 1.0 mg/L 6-BA + IAA treatment group; (**B**) 1.0 mg/L 6-BA + IBA treatment group; (**C**) 1.0 mg/L 6-BA + NAA treatment group.

Under a constant cytokinin (6-BA) concentration (0.5 or 1.0 mg/L), the addition of the same concentration (0.1 or 0.2 mg/L) of different auxins (IAA, NAA, or IBA) resulted in significant differences in both the induction rate and growth of axillary buds. As shown in Table 3, a higher concentration of 6-BA (1.0 mg/L) resulted in a greater bud induction rate compared to 0.5 mg/L. Specifically, based on this optimal concentration, the formulation with 1.0 mg/L 6-BA and 0.2 mg/L IAA was the most effective, achieving an induction rate of 86.67%. In terms of shoot morphology, buds from the 6-BA and IAA treatment were robust with dark green, smooth leaves, whereas those induced by 6-BA and IBA were relatively short despite moderate induction rates (Table 3; Figure 1A,B). The combination of 6-BA and NAA resulted in the lowest induction rate, accompanied by leaf browning and a failure of most axillary buds to sprout (Table 3; Figure 1C). Therefore, based on induction rate and shoot quality, the formulation of 2.0 g/L Hyponex supplemented with 1.0 mg/L 6-BA and 0.2 mg/L IAA was identified as optimal for axillary bud induction.

### 2.4. Shoot Proliferation with Different PGRs

**Figure 2:**
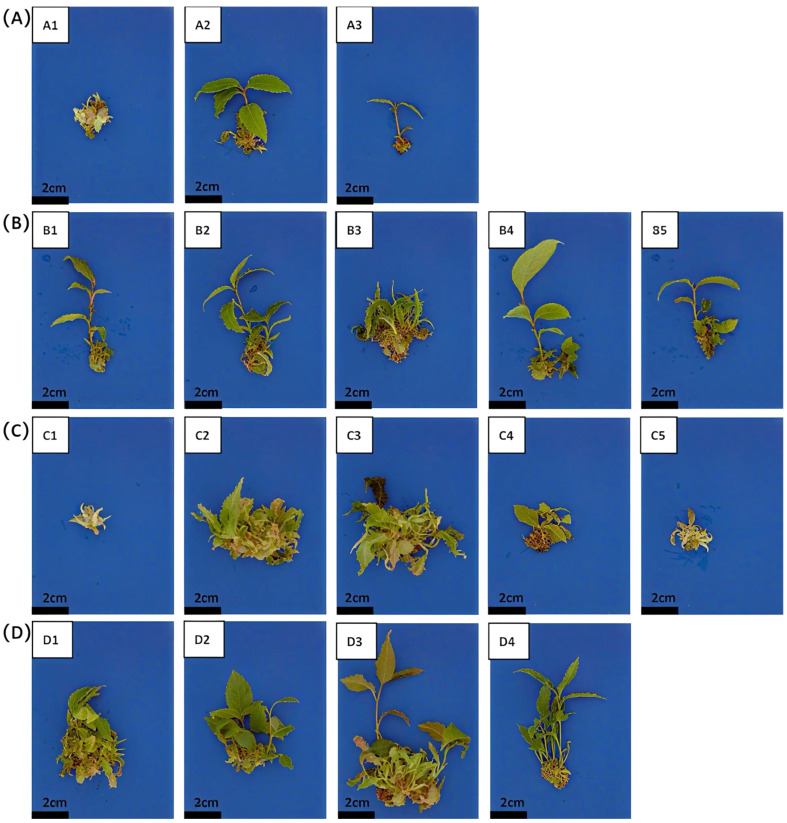
Growth induced by different hormones on bud segments. (**A**–**D**) Treatments correspond to groups (**A**–**D**) as described in Table 4. (**A**) Treatments of Group A (fixed 6-BA 1.0 mg/L + different auxins): A1: 0.1 mg/L NAA; A2: 0.1 mg/L IBA; A3: 0.1 mg/L IAA. (**B**) Treatments of Group B (fixed 6-BA 1.0 mg/L + IBA at different concentrations): B1: 0.05 mg/L; B2: 0.1 mg/L; B3: 0.15 mg/L; B4: 0.2 mg/L; B5: 0.4 mg/L. (**C**) Treatments of Group C (fixed IBA 0.15 mg/L + 6-BA at different concentrations): C1: 0.5 mg/L; C2: 1.0 mg/L; C3: 1.5 mg/L; C4: 2.0 mg/L; C5: 4.0 mg/L. (**D**) Treatments of Group D (fixed 6-BA 1.0 mg/L + IBA 0.15 mg/L + GA_3_ at different concentrations): D1: 0 mg/L; D2: 0.2 mg/L; D3: 0.5 mg/L; D4: 2.0 mg/L.

To optimize the shoot proliferation medium, we examined the effects of different concentrations and combinations of 6-BA, IBA, and GA_3_. At conventional concentrations, the combination of 6-BA and IBA proved more effective than combinations with NAA or IAA, yielding a higher proliferation coefficient (Table 4 Group A; Figure 2A). A high IBA concentration (0.4 mg/L) resulted in good proliferation and longer internodes, but the leaves exhibited wilting and yellowing in the later stages of culture. Reducing the IBA concentration to 0.15 mg/L maintained a significant proliferation effect while promoting healthier shoot growth (Table 4 Group B; Figure 2B). The optimal 6-BA concentration was determined to be 1.0 mg/L. Higher concentrations (e.g., 4.0 mg/L) inhibited shoot growth—causing stunting, discoloration, and increased mortality—despite promoting proliferation (Table 4 Group C; Figure 2C). At 1.0 mg/L, proliferation remained effective while shoots grew vigorously. Furthermore, following screening across concentrations (0.2, 0.5, and 2.0 mg/L), the addition of 0.5 mg/L GA_3_ ultimately yielded the best proliferation outcome. This was characterized by an increased shoot number, smooth leaves, purplish-red new shoots, and a maximal proliferation coefficient of 6.53 (Table 4 Group D; Figure 2D). Based on the proliferation and growth performance, the optimal proliferation medium was identified as: 2.0 g/L Hyponex + 1.0 mg/L 6-BA + 0.15 mg/L IBA + 0.5 mg/L GA_3_.

### 2.5. A Two-Step Rooting and Acclimatization Strategy

**Figure 3:**
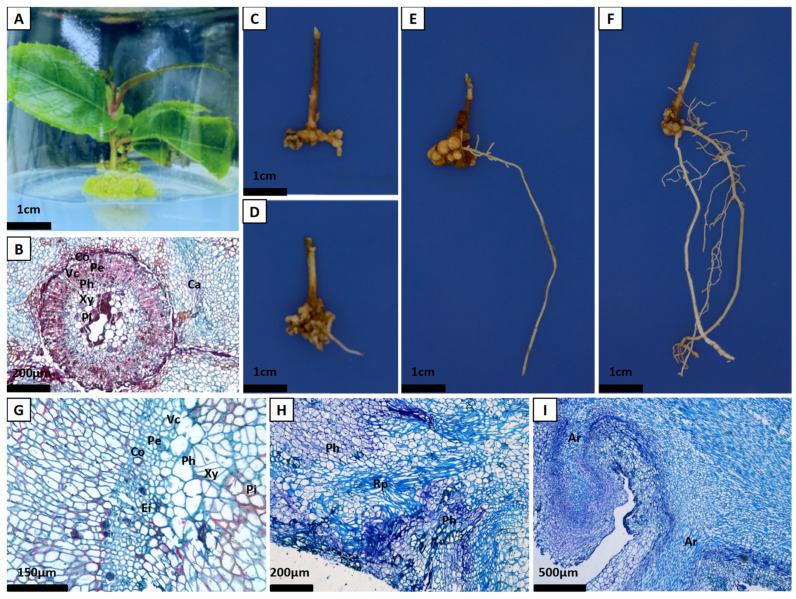
External morphology (**A**,**C**–**F**) and stem cross-sections (**B**,**G**–**I**). After pre-culture on rooting medium in vitro (**A**,**B**), and at 15 (**C**,**G**), 60 (**D**,**H**), and 75 (**E**,**I**) days post-transplantation. (**F**) shows the mature root system at 90 days. Co: Cortex; Pe: Periderm; Vc: Vascular Cambium; Xy: Xylem; Ph: Phloem; Ei: Sclerenchyma cells; Pi: Pith; Rp: Root Primordium; Ar: Adventitious Root; Ca: Callus.

Acknowledging the bottleneck of poor in vitro rooting in oil tea, we therefore developed and evaluated a novel two-step strategy combining in vitro pretreatment with ex vitro rooting. This protocol consisted of a 20-day in vitro pre-culture on an optimized medium, followed by ex vitro establishment. The pre-culture medium was formulated based on 1/2 strength Hyponex [23]. Through screening, the optimal pretreatment medium contained 1.0 mg/L NAA and 2.0 mg/L IBA. After a 20-day culture, explant bases remained flat with minimal callus (Table 5). It was noted that explants grown exclusively in vitro for 60 days showed no root emergence or primordium formation (Figure 3A,B), underscoring the limitation of purely in vitro conditions. Plantlets subjected to the two-step protocol were then dipped in 1.0 g/L ABT (a rooting powder complex) and transplanted. At 15 days post-transplantation, external base swelling and callus formation coincided with the internal dedifferentiation of bark parenchyma cells (Figure 3C,G). By 60 days, the emergence of adventitious roots corresponded to the development of distinct root primordia originating from phloem parenchyma, confirming a phloem-rooting pattern (Figure 3D,H). Subsequently, the roots underwent active elongation (75 days, Figure 3I) and matured into a fibrous system (90 days, Figure 3F). This integrated protocol achieved an 88% transplant survival rate, yielding vigorous seedlings with smooth, green leaves (Table 5).

## 3. Discussion

Effective control of microbial contamination is critical prerequisite for successfully establishing an aseptic tissue culture system in oil-tea camellia [13]. Excessive sterilization time or inappropriate disinfectant selection can easily lead to a high explant contamination rate, exacerbate browning, and even impede normal bud differentiation [24,25,26]. In plant tissue culture, the conventional combination of ethanol and mercuric chloride (HgCl_2_) is a commonly used surface sterilization method, but its efficacy is often unsatisfactory when applied to oil tea camellia. To address these limitations, improved strategies have been explored. Previous studies have shown that incorporating a preliminary running water rinse before chemical disinfection can significantly reduce the contamination burden on oil tea explants [14]. For oil-tea camellia explants, a 2–5 h tap water rinse markedly lowered contamination [15]. Furthermore, composite disinfection using both sodium hypochlorite and HgCl_2_, or the addition of novel fungicides (to control endogenous fungal contamination) and bacteriostatic agents (to control endogenous bacterium contamination) to the culture medium, has shown promise. In the cultivar ‘Haida Youcha 4’, a treatment involving 0.1% HgCl_2_ (12 min) and 2% NaClO (15 min), combined with 0.1 g/L of the bactericide in the induction medium, reduced the contamination rate to 16.7% [18]. Building upon these validated methods, the present study integrated strategies including preliminary running water rinsing, composite chemical disinfection, and the addition of a bactericide during the induction phase to systematically screen for the optimal sterilization protocol applicable to *C. chekiangoleosa* (Table 1). This integrated approach successfully minimized contamination and supported robust bud growth.

The selection of an appropriate basal medium is crucial for the efficient induction of axillary buds. Owing to its comprehensive composition, MS medium is the most widely used medium in the tissue culture of oil-tea camellia [2]. However, the present study found that for the ‘Ganhongyou 1’ cultivar of *C. chekiangoleosa*, the Hyponex medium demonstrated superior performance in bud induction, achieving an induction rate of 84.33% (Table 2). This discrepancy may be closely associated with the inorganic salt composition of the two media. MS medium is characterized by a high total concentration of inorganic salts and abundant nitrogen content [27]; In contrast, the Hyponex medium features a higher potassium content, with an N:P:K mass ratio of 7:6:19. In this study, it was observed that explants cultured on the Hyponex medium not only exhibited faster axillary bud sprouting but also developed into more robust seedlings. This may be attributed to its higher proportion of potassium (K), which plays a key role in maintaining cellular osmotic pressure, ion balance, and the buffering capacity of the medium, thereby contributing to the stability of seedling growth during the cultivation process [28]. Dai Xiaoying et al. observed a similar phenomenon in their study on axillary bud sprouting of *C. chekiangoleosa* [29]. Guo Chunxi et al. further noted that the content and ratio of N, P, and K significantly influence the growth and economic traits of oil-tea camellia, with a higher proportion of potassium being most beneficial for growth promotion [30]. In summary, the appropriate N:P:K ratio in the Hyponex medium better aligns with the nutritional requirements for bud induction and early-stage growth of ‘Ganhongyou 1’. This study also found that the concentration of Hyponex not only affects the number of axillary buds but also significantly influences their growth status, with 2.0 g/L Hyponex being most conducive to the induction and robust growth of axillary buds.

Plant growth regulators play a critical regulatory role in the induction and proliferation of axillary buds in *C. chekiangoleosa*. Existing studies have shown that in the tissue culture of oil-tea camellia, a combination of cytokinins (such as 6-BA, ZT, or TDZ) and auxins (such as NAA, IBA, or IAA) is typically employed to enhance culture efficiency, occasionally supplemented with an appropriate amount of GA_3_ or hydrolyzed casein [18,19]. This study systematically compared the effects of three auxins (IAA, IBA, and NAA) in combination with 6-BA. The results revealed that the combination of 6-BA and IAA yielded the optimal induction outcome, achieving an induction rate of 86.67% while producing buds of good quality (Table 3; Figure 1). This finding aligns with the conclusions from research on bud induction in the ‘Huashuo’ cultivar of *C. oleifera* [20]. However, responses to plant growth regulators vary among different cultivars. For instance, one study indicated that NAA plays a primary role in promoting axillary bud sprouting in the ‘Yangxin Miju’ cultivar of oil-tea camellia [31]. This highlights that genotype is a significant factor influencing hormonal effects and underscores the necessity of tailoring culture protocols according to specific varietal traits. Regarding concentration, the present study observed a positive correlation between 6-BA concentration and bud induction rate within the range of ≤1.0 mg/L (Table 3). Nevertheless, it is important to note that a higher cytokinin concentration is not always more effective. In their research on *C. chekiangoleosa*, Dai Xiaoying et al. reported that the number of induced axillary buds initially increased and subsequently decreased as the 6-BA concentration rose, suggesting that low concentrations are insufficient for effective induction, whereas high concentrations can be inhibitory. Consequently, future studies exploring higher 6-BA concentrations (>1.0 mg/L) should also focus on determining the concentration threshold of its inhibitory effects to more comprehensively optimize the induction system [29,32].

In the proliferation culture of oil-tea camellia, the cytokinin 6-BA is widely utilized, and a combination of a high concentration of 6-BA with a low concentration of auxin is generally considered an optimal formulation [18]. For example, in the ‘Xianglin 1’ cultivar, a formulation containing 5.0 mg/L 6-BA, 0.01 mg/L NAA, and 1.0 mg/L biotin achieved the highest proliferation coefficient [33]. However, excessively high concentrations of plant growth regulators can also inhibit the growth of tissue-cultured plantlets. The underlying mechanism may involve the activation of polyphenol oxidase, which in turn intensifies tissue browning and mortality [34]. The findings of this study are consistent with this observation: when the 6-BA concentration reached 4 mg/L, although the number of axillary buds was high, bud elongation was significantly inhibited, and the browning and mortality rates of the shoots increased (Figure 2; Table 4). To alleviate the dwarfing effect induced by cytokinin, GA_3_ was added to the proliferation medium in this study, leveraging its role in promoting cell elongation and division [35]. Ultimately, through the combined application of 6-BA, IBA, and GA_3_, vigorous plantlets with robust stems and dark green leaves were obtained while maintaining a high proliferation rate (Figure 2; Table 4). Similar results have also been reported in the ‘Huashuo’ and ‘Haida Youcha 4’ cultivars, where the addition of 6.0 mg/L GA_3_ significantly promoted bud proliferation and growth [33,35]. The subculture of red-flowered oil-tea camellia primarily relies on cluster shoot proliferation, which is attributed to the abundant meristems at the shoot base [29]. The continuous emergence of new shoots from the base results in uneven growth, with many exhibiting a purplish-red coloration. Independent plantlets differentiate only after the shoot height exceeds 2 cm. This phenomenon may be associated with insufficient light exposure at the base due to the relatively large leaves, consequently affecting chlorophyll synthesis and stem elongation. Therefore, it is recommended to use large-mouth vessels with ample volume and to divide the cluster shoots into smaller explants during subculture to improve light penetration, thereby enhancing shoot quality and morphology.

The in vitro rooting of oil-tea camellia plantlets presents considerable difficulty. When conventional rooting media containing NAA and IBA are used, the resulting rooted plantlets typically produce only a single main root with few fibrous roots [36]. Tang Guotao et al. conducted rooting experiments using basal media such as 1/4 MS, 1/2 MS, MS, and B5, supplemented with NAA, IBA, IAA, GA_3_, and 2,4-D. However, after numerous trials with multiple combinations, they failed to identify a suitable medium for oil-tea camellia rooting [18]. In response to the difficulty of in vitro plantlets rooting on conventional rooting media, scholars have investigated various methods to promote rooting. Among these, the “two-step method” is a common approach. It entails initially inducing the plantlets in a high-concentration auxin medium for a short period, then transferring them to a hormone-free medium to facilitate root system development. Alternatively, a bottle-free rooting method involves treating the base of the plantlets with an auxin solution and directly transplanting them for rooting [37]. Studies have shown that the conventional in-bottle rooting method often yields a low rooting rate, and the resulting root systems tend to be fragile, leading to poor transplant survival rates. In contrast, the ex vitro rooting method generally achieves a higher rooting rate, produces stronger and more robust roots, and demonstrates better transplant survival performance [38]. To address the difficulty of in vitro rooting also observed in *C. chekiangoleosa*, and considering its large, leathery leaves and sturdy stems which make the plantlets less prone to wilting during transplantation, this study adopted a “pre-treatment with auxin in vitro followed by ex vitro transplant rooting” approach, which effectively promoted rooting. This method effectively promoted rooting. It synchronizes rooting and acclimatization, is operationally simple, avoids root damage during transplantation, allows the new root system to integrate closely with the substrate, and results in a high survival rate. The concentration ratio of auxins during the pre-rooting stage is also crucial, as levels that are either too high or too low can adversely affect the rooting rate. This study found that the highest rooting rate was achieved when in vitro plantlets were cultured for 30 days in a medium supplemented with 1.0 mg/L NAA and 2.0 mg/L IBA, followed by a dip in a 1.0 g/L ABT solution (Figure 3; Table 5). This is consistent with the optimal rooting hormone ratio reported in studies on ‘Xiaoguo Youcha’ [39]. During the rooting process of white-flowered oil-tea camellia tissue culture seedlings, the addition of NAA promotes the formation and growth of primary roots, while supplementing with IBA or IAA further stimulates the development of lateral roots and enhances overall root density [40]. Furthermore, during the in-bottle pre-rooting culture stage, stems and petioles become pigmented, and the young stem tissues become more compact, achieving a higher degree of lignification within a short period. This enhances the stress tolerance of the seedlings and promotes rooting. Meanwhile, the acclimatized plantlets exhibit improved transpiration resistance, enhanced adaptability, significantly better photosynthetic performance, and stronger capacity to adapt to external environments [41], and dipping them in ABT rooting powder containing NAA and IAA as active ingredients can enhance the effectiveness of ex vitro rooting [42].

The stem serves as the most common cutting material in plant propagation, and its anatomical structure directly influences rooting capacity. Thus, the anatomical structure of the stem, along with root primordium types and rooting patterns, constitutes a key research focus in cutting anatomy [43]. The stem segment of *C. chekiangoleosa* primarily consists of the periderm, cortex, phloem, vascular cambium, xylem, and pith, with a ring of phloem fibers located between the cortex and phloem (Figure 3B) [44]. Some studies suggest that this fibrous layer physically obstructs the growth of root primordia; while others indicate that adventitious roots can still readily form if the tissue is non-continuous [45]. Therefore, this study aimed to elucidate its specific role. We found that cuttings lack preformed root primordia and possess a ring of sclerenchyma cells within the periderm. After formation, the root primordia must sequentially penetrate the phloem and breach this sclerenchymatous layer to emerge and form adventitious roots, which is likely a primary reason for their relatively long rooting cycle (approximately 60 days) (Figure 3B). Based on the timing of their formation, adventitious roots are classified as either latent or induced root primordia [46]. This study confirmed that *C. chekiangoleosa* cuttings possess no latent root primordia prior to insertion, and their adventitious roots originate entirely from primordia induced after cutting; therefore, they are classified as the induced-rooting type. These primordia originated primarily from the vascular cambium and phloem parenchyma, confirming a phloem-rooting pattern (Figure 3G–I). Furthermore, although callus formation is not directly linked to adventitious root production and occurs earlier than root primordium initiation, it plays a significant role in preventing pathogen invasion and reducing the loss of essential substances from the cuttings. Particularly during summer propagation, callus effectively inhibits cutting decay and serves as a temporary bridge for water and nutrient transport, thereby supporting the rooting process [47].

## 4. Materials and Methods

### 4.1. Plant Materials

**Figure 4:**
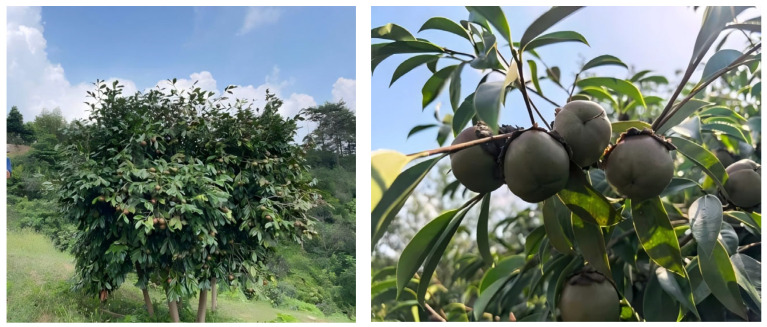
Source tree (*C. chekiangoleosa* ‘Ganhongyou 1’) at the collection site in Nanchang, Jiangxi Province.

The experimental material consisted of the provincial-level registered superior cultivar *C. chekiangoleosa* ‘Ganhongyou 1’ (Figure 4), selected by the Jiangxi Academy of Forestry. Explants were collected from healthy, semi-lignified branches with axillary buds taken from the base of superior trees located in Nanchang City, Jiangxi Province, China (28°41′ N, 115°51′ E). Tissue-cultured plantlets obtained after the initial induction of these explants were used as the study material.

### 4.2. Chemicals and Reagents

The chemicals and reagents used in this study are detailed in Table 6, which lists their full names, abbreviations, manufacturers, and specific compositions. All plant growth regulators, including 6-BA, IAA, NAA, IBA, and GA_3_, were obtained from Shanghai Zhihua Chemistry Technology Co., Ltd. (Shanghai, China). The rooting powder (ABT) was provided by Aibid Biotechnology Co., Ltd. (Beijing, China), with a total active ingredient content of 50% (20% NAA and 30% IAA). The bactericide, composed of 70% thiophanate-methyl and 30% thiram, was purchased from Zhongke Qiyuan Technology Co., Ltd. (Beijing, China), while the Hyponex fertilizer (N:P:K = 7:6:19) was sourced from Zhongda Yucheng Technology Co., Ltd. (Quanzhou, China).

### 4.3. Culture Conditions

All culture media contained 30 g/L sucrose and 7.0 g/L agar, with the pH adjusted to 5.8–6.0. Gibberellic acid (GA_3_) and the bactericide were filter-sterilized through a 0.22 μm membrane (Hangzhou Tangwei Filter Materials Co., Ltd., Hangzhou, China) and added aseptically to the medium after autoclaving, when it had cooled to 50–60 °C. The other plant growth regulators were added directly to the medium prior to autoclaving. The culture conditions were as follows: temperature, (25 ± 2) °C; light intensity, 2500 lux; and photoperiod, 14 h per day (14 h light/10 h dark cycle). For ex vitro rooting, the substrate was sprayed with a 500–600-fold dilution of 70% thiophanate-methyl wettable powder and left to stand for three days before use. Environmental conditions were controlled at 20–28 °C (day)/15–20 °C (night), 70–80% humidity, and 5000–10,000 Lux light intensity provided by 75% shading net. Irrigation was applied every 3–4 days.

### 4.4. Explant Sterilization

Newly sprouted semi-lignified shoots were collected on sunny days between June and July. After being transported to the laboratory wrapped in moist towels, the leaves were removed, and nodal segments containing axillary buds were excised. The segments were then scrubbed with a cleaning solution to remove surface contaminants, rinsed thoroughly with clean water, and subjected to seven different surface sterilization treatments. Following sterilization, damaged tissues were trimmed off, and the explants were inoculated onto the culture medium. Each treatment consisted of 30 bottles, replicated three times, with contamination and induction rates recorded ultimately. The seven disinfection methods applied were as follows: (i) Immersion in 75% ethanol for 30 s, followed by sterilization with 0.1% HgCl_2_ for 7–10 min; (ii) Immersion in 75% ethanol for 30 s, followed by sterilization with 20% sodium hypochlorite for 7–10 min; (iii) Wiping the stem segments repeatedly with 75% ethanol, rinsing under running water for 2–3 h, then immersing in 75% ethanol for 30 s, and finally sterilizing with 0.1% HgCl_2_ for 7–10 min; (iv) Hardwood branches were inserted into a cardboard box filled with moist river sand, and newly sprouted lateral buds were used as experimental materials, which were then treated according to Method iii; (v) Immersion in 75% ethanol for 30 s, rinsing with sterile water, repeating one additional ethanol immersion step, followed by sterilization with 0.1% HgCl_2_ for 7–10 min, and a final rinse with sterile water before repeating the disinfection cycle once; (vi) Immersion in 75% ethanol for 30 s, followed by sterilization with 0.1% HgCl_2_ for 7–10 min, supplemented with composite disinfection using 2.5% sodium hypochlorite for 7–10 min; and (vii) Following the disinfection procedure of Method vi, the bud induction medium was supplemented with 1 mL/L of bactericide.

### 4.5. Screening of Bud Induction Medium Concentration

The Hyponex basal medium powder, with an N-P-K ratio of 7:6:19, was used as the source of macroelements. Nodal segments with buds of ‘Ganhongyou 1’ were inoculated onto media containing Hyponex at concentrations of 0.5, 1.0, 1.5, 2.0, 2.5, and 4.0 g/L. All media were uniformly supplemented with the following components: 30 g/L sucrose as the carbon source, 7 g/L agar as the solidifying agent, and 1.0 mg/L 6-BA along with 0.01 mg/L NAA as plant growth regulators [18,19]. Each culture bottle was inoculated with one nodal segment. Each treatment included 30 bottles and was replicated three times. The axillary bud germination rate was assessed after 30 days of culture. The axillary bud induction rate was calculated as the percentage of segments with sprouted axillary buds relative to the total number of inoculated segments.

### 4.6. Optimization of PGR Combinations for Bud Induction

Using the modified Hyponex medium (2 g/L) as the basal medium, a total of twelve different plant growth regulator combinations were established, involving 6-BA (0.5, 1.0 mg/L), IAA (0, 0.1, 0.2 mg/L), IBA (0, 0.1, 0.2 mg/L), and NAA (0, 0.1, 0.2 mg/L). All media were supplemented with 30 g/L sucrose and 7 g/L agar, with the pH adjusted to 5.6. Following initial disinfection, the explants were inoculated, placing one nodal segment per culture bottle. Each treatment consisted of 30 bottles and was replicated three times. The axillary bud germination rate was recorded after 30 days of culture.

### 4.7. Shoot Proliferation Culture

Aseptic shoots from the initial culture were proliferated on the modified Hyponex medium. The experimental comprised four groups (A–D) designed to screen for optimal auxin type, auxin concentration, 6-BA concentration, and the best overall proliferation medium combination. Each treatment included five bottles. Cultures were initiated with two axillary buds per bottle and subcultured three times using four shoot clusters per bottle. The proliferation coefficient (final shoot number/initial shoot number) was recorded after 30 days of the third subculture cycle when growth had stabilized; shoot growth status was concurrently observed.

### 4.8. Rooting Pre-Treatment and Ex Vitro Rooting

Following proliferation, axillary shoots (3–4 cm tall) were selected, excised at the base, and transferred to a pre-rooting medium based on 1/2-strength Hyponex medium. The experiment comprised nine different plant growth regulator combinations, involving NAA (0.5, 1.0, 2.0 mg/L) and IBA (0.5, 1.0, 2.0 mg/L). For each treatment, 10 shoots were inoculated per bottle, with five bottles per replicate and three replicates. After 30 days, plantlets were removed, and the medium at the base was carefully washed off. The basal ends were then dipped in a 1.0 g/L ABT solution before being transplanted into seedling bags (6 cm × 8 cm) containing a mixture of 65% peat, 10% perlite, 10% coconut coir, 10% rice husks, and 5% plant ash. After transplanting, the substrate was thoroughly watered. A fungicide (Thiophanate-methyl, Hulian Bio-Pharmaceutical (Xiayi) Co., Ltd., Shanghai, China) was applied, and the plantlets were covered with a plastic film to maintain humidity. The film was periodically removed for ventilation. The transplant survival rate, defined as the percentage of plantlets that developed new roots relative to the total number transplanted, was assessed after two months.

### 4.9. Data Analysis

All data were subjected to one-way analysis of variance (ANOVA) using SPSS19.0. The assumption of homogeneity of variances was verified using Levene’s test. When a significant overall effect was detected (*p* < 0.05), Tukey’s honestly significant difference (HSD) post hoc test was applied for multiple comparisons among treatment means. Data in tables are presented as the mean ± standard deviation (SD). Different lowercase letters indicate statistically significant differences at *p* < 0.05 among treatments. The primary dataset from all screening experiments is provided in Table S1.

## 5. Conclusions

This study successfully established a complete and efficient micropropagation system for the provincial-level registered superior cultivar *C. chekiangoleosa* ‘Ganhongyou 1’. We systematically optimized the key stages: bud induction (86.67% rate on a medium of 2.0 g/L Hyponex, 1.0 mg/L 6-BA, and 0.2 mg/L IAA), shoot proliferation (6.53 proliferation coefficient with 2 g/L Hyponex, 1.0 mg/L 6-BA, 0.15 mg/L IBA, and 0.5 mg/L GA_3_), and rooting–acclimatization via an innovative two-step strategy (in vitro pre-culture + ex vitro treatment), which achieved an 88% transplant survival rate. Anatomical observations further elucidated the rooting mechanism: *C. chekiangoleosa* exhibits an induced-rooting type, with root primordia originating mainly from phloem parenchyma cells adjacent to the vascular cambium, representing a typical phloem-rooting pattern. This system effectively addresses common challenges in oil-tea camellia tissue culture, such as high contamination rates, severe browning, and rooting difficulties, thereby providing a reliable technical foundation for the large-scale production of this valuable species.

## Figures and Tables

**Table 1:** The effect of different sterilization methods on the surface sterilization of explants.

Method	Disinfection Treatment	Pollution Rate (%)	Induction Rate (%)	Shoot Grade
i	75% C_2_H_5_OH (30 s) + 0.1% HgCl_2_ (7–10 min)	91.00 ± 1.73 a	1.00 ± 1.73 f	Mainly Grade III and IV
ii	75% C_2_H_5_OH (30 s) + 20% NaClO (7–10 min)	95.67 ± 2.31 a	1.00 ± 1.73 f	Mainly Grade III and IV
iii	Pretreatment + 75% C_2_H_5_OH (30 s) + 0.1% HgCl_2_ (7–10 min)	51.00 ± 1.73 b	15.67 ± 2.31 e	Mainly Grade II and III
iv	Indoor Cultivation + Pretreatment + 75% C_2_H_5_OH (30 s) + 0.1% HgCl_2_ (7–10 min)	41.00 ± 7.21 c	25.33 ± 4.04 d	Mainly Grade II and III
v	Pretreatment + 75% C_2_H_5_OH (30 s) + 0.1% HgCl_2_ (7–10 min) + 0.1% HgCl_2_ (7–10 min)	38.00 ± 1.73 c	32.00 ± 1.73 c	Mainly Grade II and III
vi	Pretreatment + 75% C_2_H_5_OH (30 s) + 0.1% HgCl_2_ (7–10 min) + 20% NaClO (7–10 min)	24.67 ± 4.04 d	54.67 ± 4.04 b	Mainly Grade I and II
vii	Pretreatment + 75% C_2_H_5_OH (30 s) + 0.1% HgCl_2_ (7–10 min) + 20% NaClO (7–10 min) + 1 mL/L Bactericide	18.00 ± 1.73 e	62.33 ± 4.04 a	Mainly Grade I and II

Different lowercase letters in the same column indicate significant differences (*p* < 0.05). Shoots were graded into four categories: Grade I (>4 leaves, obvious internodes, length ≥ 3.0 cm); Grade II (3 unfolded leaves, 0.8 cm ≤ length < 3.0 cm); Grade III (swollen buds, no leaf unfolding); Grade IV (no change or browning/death) (Figure S1). Bud length was measured from the leaf axil to the bud tip.

**Table 2:** The effects of different Hyponex concentrations on the induction of axillary buds.

No.	Improve Hyponex (g/L)	Induction Rate (%)
1	0.5	17.33 ± 5.69 d
2	1.0	37.00 ± 7.00 c
3	1.5	60.67 ± 2.52 b
4	2.0	84.33 ± 5.03 a
5	2.5	60.33 ± 2.89 b
6	4.0	12.33 ± 3.06 d

Different lowercase letters in the same column indicate significant differences (*p* < 0.05).

**Table 3:** The effects of different combinations of hormones on the induction of axillary buds in culture.

No.	6-BA (mg/L)	IAA (mg/L)	IBA (mg/L)	NAA (mg/L)	Induction Rate (%)
1	0.5	0.1	0	0	50.67 ± 6.03 cd
2	1.0	0.1	0	0	64.33 ± 5.13 b
3	0.5	0.2	0	0	62.33 ± 5.03 b
4	1.0	0.2	0	0	86.67 ± 3.51 a
5	0.5	0	0.1	0	63.00 ± 6.93 b
6	1.0	0	0.1	0	65.33 ± 6.81 b
7	0.5	0	0.2	0	41.00 ± 3.46 de
8	1.0	0	0.2	0	57.67 ± 8.02 bc
9	0.5	0	0	0.1	10.33 ± 2.52 g
10	1.0	0	0	0.1	29.00 ± 8.54 f
11	0.5	0	0	0.2	34.00 ± 11.53 f
12	1.0	0	0	0.2	41.33 ± 5.13 de

Different lowercase letters in the same column indicate significant differences (*p* < 0.05). 6-benzylaminopurine (6-BA); indole-3-acetic acid (IAA); indole-3-butyric acid (IBA); 1-naphthaleneacetic acid (NAA).

**Table 4:** The effects of different combinations of hormones on the proliferation of axillary buds in culture.

Group		Hormone	Proliferation Coefficient	Growth Condition
A	A1	1.0 mg/L 6-BA + 0.1 mg/L NAA	1.43 ± 0.21 h	Bud number was limited, with signs of mortality observed.
	A2	1.0 mg/L 6-BA + 0.1 mg/L IBA	3.70 ± 0.56 h	The buds were numerous and fine.
	A3	1.0 mg/L 6-BA + 0.1 mg/L IAA	1.80 ± 0.36 fgh	Bud number was limited, with signs of mortality observed.
B	B1	1.0 mg/L 6-BA + 0.05 mg/L IBA	1.97 ± 0.15 efgh	Bud number was limited.
	B2	1.0 mg/L 6-BA + 0.1 mg/L IBA	3.43 ± 0.40 d	Bud number was moderate.
	B3	1.0 mg/L 6-BA + 0.15 mg/L IBA	4.00 ± 0.56 b	The buds were numerous and the new shoots were purplish-red.
	B4	1.0 mg/L 6-BA + 0.2 mg/L IBA	3.60 ± 0.46 d	Bud number was moderate.
	B5	1.0 mg/L 6-BA + 0.4 mg/L IBA	2.50 ± 0.20 ef	Bud number was limited.
C	C1	0.5 mg/L 6-BA + 0.15 mg/L IBA	2.47 ± 0.51 efg	Bud number was limited.
	C2	1.0 mg/L 6-BA + 0.15 mg/L IBA	4.80 ± 0.20 b	The buds were numerous and the new shoots were purplish-red.
	C3	1.5 mg/L 6-BA + 0.15 mg/L IBA	4.67 ± 0.59 bc	The buds were numerous and the new shoots were purplish-red.
	C4	2.0 mg/L 6-BA + 0.15 mg/L IBA	2.70 ± 0.46 e	The buds were numerous but fine, and the leaves were severely chlorotic and necrotic.
	C5	4.0 mg/L 6-BA + 0.15 mg/L IBA	1.67 ± 0.42 gh	The buds were numerous but fine, and the leaves were severely chlorotic and necrotic.
D	D1	1.0 mg/L 6-BA + 0.15 mg/L IBA	4.93 ± 0.75 b	The buds were numerous and the new shoots were purplish-red.
	D2	1.0 mg/L 6-BA + 0.15 mg/L IBA + 0.2 mg/L GA_3_	4.00 ± 0.20 cd	The buds were numerous and the new shoots were purplish-red.
	D3	1.0 mg/L 6-BA + 0.15 mg/L IBA + 0.5 mg/L GA_3_	6.53 ± 0.55 a	Significant proliferation with dense, purplish-red buds and smooth leaf surfaces.
	D4	1.0 mg/L 6-BA + 0.15 mg/L IBA + 2.0 mg/L GA_3_	1.87 ± 0.35 fgh	Bud number was limited.

Different lowercase letters in the same column indicate significant differences (*p* < 0.05). 6-benzylaminopurine (6-BA); indole-3-acetic acid (IAA); indole-3-butyric acid (IBA); 1-naphthaleneacetic acid (NAA); gibberellic acid (GA_3_).

**Table 5:** Pre-rooting treatment for seedling conditioning before transplanting.

No.	Hormone (mg/L)		Transplant Survival Rate (%)	Growth Condition
	NAA	IBA		
1	0.5	1.0	16.00 ± 4.00 e	The majority of the plants withered and died.
2	0.5	2.0	57.33 ± 8.33 c	The stems exhibited elongation with small and yellow leaves.
3	0.5	4.0	53.33 ± 10.07 b	The stems exhibited elongation with large, smooth, and tender green leaves.
4	1.0	1.0	37.33 ± 6.11 d	No stem elongation was observed, and the leaves were small and chlorotic.
5	1.0	2.0	88.00 ± 4.00 a	The stems exhibited elongation with large, smooth, and dark green leaves.
6	1.0	4.0	76.00 ± 4.00 ab	The stems exhibited elongation with large, smooth, and green leaves.
7	2.0	1.0	37.33 ± 6.11 d	No stem elongation was observed, and the leaves were small and chlorotic.
8	2.0	2.0	60.00 ± 8.00 c	The stems exhibited elongation with small, tender green leaves.
9	2.0	4.0	53.33 ± 10.07 c	The stems exhibited elongation with small and chlorotic leaves.

Different lowercase letters in the same column indicate significant differences (*p* < 0.05). Indole-3-butyric acid (IBA); 1-naphthaleneacetic acid (NAA).

**Table 6:** Details of the Chemicals and Reagents Used.

Full Term	Abbreviation	Brand and Ingredient Specifications
Hyponex [48,49,50]		Zhongda Yucheng Technology Co., Ltd., Quanzhou, China. N:P:K = 7:6:19.
ABT Rooting Powder	ABT	Aibidi Biotechnology Co., Ltd., Beijing, China. A soluble powder containing 50% total active ingredients as a mixture of 20% 1-naphthaleneacetic acid (NAA) and 30% indole-3-acetic acid (IAA).
Bactericide		Zhongke Qiyuan Technology Co., Ltd., Beijing, China Active ingredients: 70% thiophanate-methyl and 30% thiram (by weight).
Thiophanate-methyl		Hulian Bio-Pharmaceutical (Xiayi) Co., Ltd., Shanghai, China
6-Benzylaminopurine	6-BA	Zhihua Chemistry Technology Co., Ltd., Shanghai, China
Indole-3-acetic acid	IAA	Zhihua Chemistry Technology Co., Ltd., Shanghai, China
1-Naphthaleneacetic acid	NAA	Zhihua Chemistry Technology Co., Ltd., Shanghai, China
3-Indolebutyric acid	IBA	Zhihua Chemistry Technology Co., Ltd., Shanghai, China
Gibberellic acid	GA_3_	Zhihua Chemistry Technology Co., Ltd., Shanghai, China

## Data Availability

The data presented in this study are available within the article.

## Supplementary Materials

The following supporting information can be downloaded at: https://www.mdpi.com/article/10.3390/plants15060871/s1, Table S1: Primary dataset from all screening experiments. Figure S1: Morphological characteristics of axillary buds at different germination grades.

## References

[1] Gao C., Yang R., Yuan D. (2017). Characteristics of Developmental Differences between Fertile and Aborted Ovules in *Camellia oleifera*. J. Am. Soc. Hortic. Sci..

[2] Zhang M., Wang A., Qin M., Qin X., Yang S., Su S., Sun Y., Zhang L. (2021). Direct and Indirect Somatic Embryogenesis Induction in *Camellia oleifera* Abel. Front. Plant Sci..

[3] Shen T., Huang B., Xu M., Zhou P., Ni Z., Gong C., Wen Q., Cao F., Xu L.-A. (2022). The Reference Genome of *Camellia chekiangoleosa* Provides Insights into *Camellia* Evolution and Tea Oil Biosynthesis. Hortic. Res..

[4] Wei T., Dong L., Zhong S., Jing H., Deng Z., Wen Q., Li J. (2022). Chemical Composition of *Camellia chekiangoleosa* Hu. Seeds during Ripening and Evaluations of Seed Oils Quality. Ind. Crops Prod..

[5] Cicero A.F.G., Derosa G., Pisciotta L., Barbagallo, C., on behalf of the SISA-PUFACOL Study Group (2015). Testing the Short-Term Efficacy of a Lipid-Lowering Nutraceutical in the Setting of Clinical Practice: A Multicenter Study. J. Med. Food.

[6] Sun Y., Gao L., Hou W., Wu J. (2020). *β*-Sitosterol Alleviates Inflammatory Response via Inhibiting the Activation of ERK/P38 and NF-*κ* B Pathways in LPS-Exposed BV2 Cells. BioMed Res. Int..

[7] Wang Z.-X., Wang Y.-Y. (2014). Evaluation of the Provincial Competitiveness of the Chinese High-Tech Industry Using an Improved TOPSIS Method. Expert Syst. Appl..

[8] Zhong S., Huang B., Wei T., Deng Z., Li J., Wen Q. (2023). Comprehensive Evaluation of Quality Characteristics of Four Oil-Tea Camellia Species with Red Flowers and Large Fruit. Foods.

[9] Bednarek P.T., Orłowska R. (2020). Plant Tissue Culture Environment as a Switch-Key of (Epi) Genetic Changes. Plant Cell Tissue Organ Cult. (PCTOC).

[10] Ho T.-T., Murthy H.N., Park S.-Y. (2020). Methyl Jasmonate Induced Oxidative Stress and Accumulation of Secondary Metabolites in Plant Cell and Organ Cultures. Int. J. Mol. Sci..

[11] Isah T., Umar S., Mujib A., Sharma M.P., Rajasekharan P.E., Zafar N., Frukh A. (2018). Secondary Metabolism of Pharmaceuticals in the Plant in Vitro Cultures: Strategies, Approaches, and Limitations to Achieving Higher Yield. Plant Cell Tissue Organ Cult..

[12] Loyola-Vargas V.M., Ochoa-Alejo N., Loyola-Vargas V.M., Ochoa-Alejo N. (2018). An Introduction to Plant Tissue Culture: Advances and Perspectives. Plant Cell Culture Protocols.

[13] Garg M., Datta S., Ahmad S., Anis M., Khanam M.N. (2024). Plant Tissue Culture: A Potential Tool for the Production of Secondary Metabolites. Vitro Propagation and Secondary Metabolite Production from Medicinal Plants: Current Trends (Part 2).

[14] Phillips G.C., Garda M. (2019). Plant Tissue Culture Media and Practices: An Overview. Vitr. Cell. Dev. Biol. Plant.

[15] Zhang H., Han M., Nie X., Fu X., Hong K., He D. (2024). Production of *Camellia oleifera* Abel Seed Oil for Injection: Extraction, Analysis, Deacidification, Decolorization, and Deodorization. Foods.

[16] Ma Y., Xu J., Qi J., Zhao D., Jin M., Wang T., Yang Y., Shi H., Guo L., Zhang H. (2024). Crosstalk among Plant Hormone Regulates the Root Development. Plant Signal. Behav..

[17] Iqbal M., Wali V.K., Bakshi P., Kour K., Razdan V.K., Sinha B.K., Sood K.K. (2019). In Vitro Propagation of Citrus Species through Callus Induction and Regeneration: A Review. Int. J. Curr. Microbiol. Appl. Sci..

[18] Liu J.P., Hu H.Y., Wu W.Q., Huang X.L., Huang D.Y., Wang J., Lai H.G. (2024). Research progress of oil-tea *Camellia* culture in vitro. Nonwood For. Res..

[19] Yang S.X., Luo S.Q., Yang Z.N., Quan W.X., Ding Q., Wang K.F., Wang Y. (2024). Research Progress in Tissue Culture of *Camellia oleifera*. Mol. Plant Breed..

[20] Li Z., Tan X.F., Yuan J., Lu K., Zhang L., Lin Q., Lyu J.B. (2014). Tissue Culture and Highly Efficient Rooting of *Camellia oleifera* ‘Huashuo’. Plant Physiol. J..

[21] Yuan D.Y., Fan X.M., Tan X.F., Zeng Y.L., Tang J., Yang Y. (2013). Culture in vitro and rapid propagation techniques of buds and leaves in *Camellia oleifera*. J. Nanjing For. Univ..

[22] Dai X.Y., Gan R., Cheng Q.Q., Song X.C., Xiao F.M. (2015). Rooting techniques of tissue culture seedlings from *Camellia oleifera*. South. For. Sci..

[23] An J., Kim P.B., Park H.B., Kim S., Park H.J., Lee C.W., Lee B.-D., Kim N.Y., Hwang J.E. (2021). Effects of Different Growth Media on In Vitro Seedling Development of an Endangered Orchid Species *Sedirea japonica*. Plants.

[24] Nguyen H.T., Khuat Q.V., Ninh T.T., Dang A.T.P., Nguyen L.T., Kalasnıkova E.A., Batukaev A.A., Kirakosyan R.N. (2025). Direct Organogenesis of *Epipremnum aureum* G.S. Bunting for Mass Propagation. Plants.

[25] Zhang C., Van Der Heijden M.G.A., Dodds B.K., Nguyen T.B., Spooren J., Valzano-Held A., Cosme M., Berendsen R.L. (2024). A Tripartite Bacterial-Fungal-Plant Symbiosis in the Mycorrhiza-Shaped Microbiome Drives Plant Growth and Mycorrhization. Microbiome.

[26] Zhang G., Sajjad M., Pu S., Song C., Luo F., Luo K., Xu Y., Zhang H., Zheng Y. (2025). Establishing a Leaf-Derived Tissue Culture and Rapid Propagation Method for Red Fruit Ginseng. Horticulturae.

[27] Reddy J. (2023). Plant Tissue Culture.

[28] Stricker J.L., Adragna N.C., Lauf P.K. (2018). Cytosolic Internalization of the Na/K ATPase Ouabain Receptor Complex (NORC). FASEB J..

[29] Dai X.Y., Gong L., Luo C.L., Cheng Q.Q., Liu X.L. Rapid propagation technology of *Camellia chekiangoleosa*. Nonwood For. Res..

[30] Guo C.X., Yang X.J., Hu Y.L., Yang S.Y., Pan Z.F., Long X.Y. (2024). Effects of Amino Acids and Different Nnutrient Elements on the Growth and Economic Characters of *Camellia oleifera*. Mol. Plant Breed..

[31] Zhang W.N. (2013). Tissue Culture and Rapid Propagation of *Camellia oleifera* ‘Yangxin Micha’. Master’s Thesis.

[32] Gong Z., Wang H.F., Zhang H., He C.M., Xu Q.L. (2015). Tissue Culture and Rapid Propagation of *Camelliasemiserrata*. J. Guangdong For. Sci. Technol..

[33] Wang D., Chen Y., Wang X., Peng S., Chen L., Ma L., Luo J., Yang X. (2015). Establishment of High Efficient Propagation System of Tissue Culture Seedling of *Camellia oleifera*. J. Cent. S. Univ. For. Technol..

[34] Arora V., Ghosh M.K., Pal S., Gangopadhyay G. (2017). Allele Specific CAPS Marker Development and Characterization of Chalcone Synthase Gene in Indian Mulberry (*Morus* spp., Family Moraceae). PLoS ONE.

[35] Zhang Q.F., Chen J.M., Hou X.X., Li S.Y., Wang J., Lai H.G. (2022). In vitro rapid propagation of *Camellia vietnamensis* Haida youcha 4 and nurse seed grafting using its tissue culture bud as scion. Chin. J. Oil Crop Sci..

[36] Li Z., Tan X., Liu Z., Lin Q., Zhang L., Yuan J., Zeng Y., Wu L. (2016). In Vitro Propagation of *Camellia oleifera* Abel. Using Hypocotyl, Cotyledonary Node, and Radicle Explants. HortScience.

[37] Li S., Zhao R., Ye T., Guan R., Xu L., Ma X., Zhang J., Xiao S., Yuan D. (2022). Isolation, Purification and PEG-Mediated Transient Expression of Mesophyll Protoplasts in *Camellia oleifera*. Plant Methods.

[38] Lin Z., Huang L.-J., Yu P., Chen J., Du S., Qin G., Zhang L., Li N., Yuan D. (2023). Development of a Protoplast Isolation System for Functional Gene Expression and Characterization Using Petals of *Camellia oleifera*. Plant Physiol. Biochem..

[39] Ye C., He Z., Peng J., Wang R., Wang X., Fu M., Zhang Y., Wang A., Liu Z., Jia G. (2023). Genomic and Genetic Advances of Oiltea-Camellia (*Camellia oleifera*). Front. Plant Sci..

[40] Luan F., Zeng J., Yang Y., He X., Wang B., Gao Y., Zeng N. (2020). Recent Advances in Camellia Oleifera Abel: A Review of Nutritional Constituents, Biofunctional Properties, and Potential Industrial Applications. J. Funct. Foods.

[41] Zhang F., Zhu F., Chen B., Su E., Chen Y., Cao F. (2022). Composition, Bioactive Substances, Extraction Technologies and the Influences on Characteristics of *Camellia oleifera* Oil: A Review. Food Res. Int..

[42] Quan W., Wang A., Gao C., Li C. (2022). Applications of Chinese *Camellia oleifera* and Its By-Products: A Review. Front. Chem..

[43] Ausari P.K., Soni N., Kanpure R.N., Ninama N., Bhandari J. (2023). Effect of Indole-3-Butyric Acid (IBA) on Hardwood Cutting of Grapes (*Vitis vinifera* L.) Cv. Pusa Navrang. Int. J. Environ. Clim. Change.

[44] Li X., Wang R., Zhou B., Wang X., Wang J., Zhao M., Li C. (2022). Characterization of Root Morphology and Anatomical Structure of Spring Maize under Varying N Application Rates and Their Effects on Yield. Agronomy.

[45] Atucha A., Workmaster B.A., Bolivar-Medina J.L. (2021). Root Growth Phenology, Anatomy, and Morphology among Root Orders in *Vaccinium macrocarpon* Ait. Botany.

[46] Pandey S., Husen A. (2022). Role of Plant Stem or Shoot Cutting Positions and Hormone Treatments in Adventitious Root Formation. Environmental, Physiological and Chemical Controls of Adventitious Rooting in Cuttings.

[47] Stokes C., Bassuk N., Miller B. (2023). Light Reduction, Banding, and IBA Treatments Influence Adventitious Rooting of Lindera Benzoin Stem Cuttings. HortScience.

[48] Hwang J.E., Park H.B., Tho J.-H., Kim M., Park H.J., Kim S., Lee C.W., Kim Y.-J. (2025). Influence of Basal Medium and Organic Additives on In Vitro Germination and Plant Growth of Endangered Orchid Gastrochilus Fuscopunctatus. Plants.

[49] Do V.N.T., Hsu S.-T., Lee Y.-I. (2019). Clonal Propagation In Vitro of Paphiopedilum Hybrids from Adult Plants. HortScience.

[50] Wu K., Zeng S., Lin D., Teixeira Da Silva J.A., Bu Z., Zhang J., Duan J. (2014). In Vitro Propagation and Reintroduction of the Endangered Renanthera Imschootiana Rolfe. PLoS ONE.

