# Supplementary material for: An Efficient Micropropagation Protocol for Camellia chekiangoleosa ‘Ganhongyou 1’ via Stem Segment Culture
Source: Plants (Basel). 2026 Mar 11;15(6):871. doi: 10.3390/plants15060871 (PMC13030357; doi:10.3390/plants15060871)
Supplement: Supplementary file 1 [file plants-15-00871-s001.zip › Figure S1 Morphological characteristics of axillary buds at different germination grades..pdf]

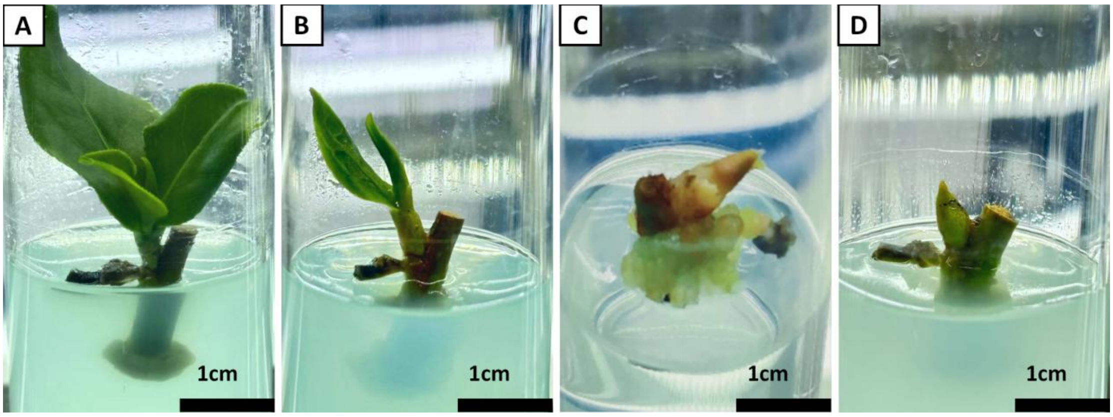

Figure S1. Morphological characteristics of axillary buds at different germination grades. **(A)** Grade I (>4 leaves, obvious internodes, length ≥ 3.0 cm) ; **(B)** Grade II (3 unfolded leaves, 0.8 cm ≤ length < 3.0 cm); **(C)** Grade III (swollen buds, no leaf unfolding); **(D)** Grade IV (no change or browning/death).
